# Analysis of the current status of rehabilitation motivation and its influencing factors in older adults with stroke: a cross-sectional study

**DOI:** 10.3389/fnagi.2023.1186681

**Published:** 2023-04-27

**Authors:** Mingyang Tan, Hongyu Li, Xiuli Wang

**Affiliations:** ^1^Department of Nursing, Jinzhou Medical University, Jinzhou, China; ^2^Department of Radiotherapy, The First Affiliated Hospital of Jinzhou Medical University, Jinzhou, China

**Keywords:** stroke, influencing factors, rehabilitation, cross-sectional survey, correlation

## Abstract

**Objective:**

Among stroke patients, exercise adherence is an important factor in reducing the rate of physical disability and mortality. Rehabilitation exercise after stroke is a safe and effective measure to restore normal body functions, but the analysis of factors influencing patients’ motivation for rehabilitation is not well established. Therefore, this study will explore the influencing factors of rehabilitation motivation in older adults with stroke so as to reduce the disability rate of stroke.

**Method:**

A convenience sampling method was used to study 350 patients in a stroke ward of a tertiary care hospital in Jinzhou, Liaoning Province. Patients’ general demographic data, Multidimensional Scale of Perceived Social Support (PSSS), Questionnaire of Exercise Adherence (EAQ), Tampa scale of kinesiophobia (TSK-11), and Motivation in stroke patients for rehabilitation scale (MORE) were assessed. ANOVA or t-test analysis, correlation analysis, and linear regression analysis were used to explore the factors influencing the motivation for rehabilitation in older adults with stroke.

**Results:**

The results showed that stroke patients’ motivation for rehabilitation was at a moderate level. Perceived social support, exercise adherence, and stroke motivation were positively correlated (*r* = 0.619, *p* < 0.01; *r* = 0.569, *p* < 0.01), and kinesiophobia was negatively correlated with stroke motivation (*r* = −0.677, *p* < 0.01). Time of stroke, location of the lesion, perceived social support, exercise adherence, and kinesiophobia are influential factors affecting patients’ motivation to recover.

**Conclusion:**

In the rehabilitation program for older adult patients with stroke, healthcare providers should specify targeted rehabilitation measures according to the different degrees of the patient’s condition, so as to improve the effectiveness of rehabilitation medical implementation.

## 1. Introduction

Stroke is a common disease that threatens human health and life. It is a sudden rupture or cerebrovascular obstruction caused by a variety of factors, including ischemic stroke and hemorrhagic stroke. It is characterized by high incidence, recurrence, disability, and mortality (Jadhav et al., 2021). In recent years, the incidence of older adult patients with stroke has shown an increasing trend, which has brought a serious impact on the normal work and life of patients and posed a great threat to their life and health safety (GBD 2019 Stroke Collaborators, 2021). One study showed that the risk of stroke in China is 39.9%, which is the highest in the world (Ma et al., 2021). Stroke patients are often accompanied by different degrees of functional impairment, such as depression, swallowing disorder, perceptual impairment, and cognitive impairment (Das and Rajanikant, 2018; Jones et al., 2020; Kim et al., 2020; Tater and Pandey, 2021), all of which will seriously affect the future quality of life of patients. Rehabilitation therapy is currently the primary measure to reduce the rate of disability (Isaacs-Itua and Wong, 2021) and is a safe intervention (Saunders et al., 2020). Therefore, early, timely, and effective rehabilitation exercises are particularly important.

Rehabilitation is a broad term that encompasses not only specialized intervention methods for specific injuries but also the process of providing a range of rehabilitative exercise care by the rehabilitation team (Shaw et al., 2020). However, the current global state of rehabilitation is not promising, with systems for rehabilitation time, rehabilitation content, rehabilitation programs, and rehabilitation personnel not yet in place between different countries, resulting in a uniform normative standard of behavior for rehabilitation that has not yet been developed. Some studies have shown (Kwok et al., 2012; Bates et al., 2013; Lam Wai Shun et al., 2017) that factors such as medical personnel, family members, and patients themselves can influence the rehabilitation outcome, especially the patients’ own cognitive and attitudinal factors are extremely important for the rehabilitation outcome. Evidence-based data show (Maggio et al., 2019) that motivation for rehabilitation plays a dominant role in proactivity in the rehabilitation process, and that a strong willingness to rehabilitate can promote patients to actively seek knowledge about rehabilitation exercises, increase their awareness of rehabilitation, and reduce the disability rate. This is also true for post-stroke patients, and patients’ motivation should be enhanced so that it can be translated into a willingness to rehabilitate in daily life.

Social support is an influential factor that predicts patient motivation to recover (Lee and Won, 2022). Studies have shown that effective social support can not only effectively alleviate patients’ anxiety, depression, and other negative emotions in the rehabilitation process, but also effectively improve patients’ physiological and psychological health (Pérez-Rojas and del Pilar Torres-Arreola, 2012), increase their compliance with functional exercise (Zhang et al., 2020), promote disease recovery and reduce the disability rate. However, the current social support is not comprehensive and systematic, which makes most stroke patients fail to obtain effective health service resources in time after the onset of the disease and are unable to understand the disease correctly, which in turn aggravates the patients’ fear of the disease (Mahamid et al., 2021). Kinesiophobia is a common problem for rehabilitation patients. Kinesiophobia patients avoid training due to exercise fear, which hinders the rehabilitation process. Studies have shown that kinesiophobia can make patients fear rehabilitation exercise, causing mental health problems such as fear and worry, and even disuse syndrome in severe cases (Saka et al., 2021; Shekhar et al., 2022).

Currently, studies on factors influencing stroke rehabilitation have received widespread attention at home and abroad. However, fewer studies have been conducted to analyze the influencing factors of motivation for post-stroke rehabilitation. Therefore, this study will assess the level of motivation for rehabilitation in older adult patients with stroke and comprehensively analyze its influencing factors in order to improve patients’ own compliance to participate in rehabilitation, continuously improve the clinical work of health care professionals to improve the implementation rate of rehabilitation and reduce the rate of disability after stroke. This study proposes the following hypothesis: perceived social support, exercise adherence, and kinesiophobia are the influential factors affecting rehabilitation motivation in older adult patients with stroke.

## 2. Method

### 2.1. Participant

In this study, a cross-sectional survey was used to collect 350 patients from January to March 2023 in the stroke rehabilitation unit of a tertiary care hospital in Jinzhou, Liaoning Province. Inclusion criteria: (1) patients diagnosed with stroke according to the relevant regulations of the American Stroke Association (Sacco et al., 2013); (2) Age ≥ 60 years; (3) able to communicate normally and conscious, by the Short Portable Mental Status Questionnaire (SPMSQ) (Short Pfeiffer, 1975); (4) Informed consent, voluntary participation in this study. Exclusion criteria: (1) depression or other major diseases; (2) patients or family members refused to participate; (3) those who withdrew in the middle of the study. This study was approved by the Medical Ethics Committee of Jinzhou Medical University.

### 2.2. Evaluation scale

#### 2.2.1. The general information questionnaire

Patient’s gender, age, education, time of stroke, lesion location, stroke type, and whether they had health insurance were collected.

#### 2.2.2. Multidimensional scale of perceived social support

Developed by Ziment et al (1990), and currently, the most used version is a revised version by Chinese scholar Jiang (2009), the scale has a total of 12 items and measures only perceived social support, including perceived family, friends, and other support, with four questions for each dimension, using a seven-point Likert scale. The higher the score, the higher the patient’s perceived social support. The Cronbach’s alpha of the scale in this study was 0.984.

#### 2.2.3. Questionnaire of exercise adherence

This questionnaire was developed by Lin et al. (2013) and includes three dimensions with 14 entries. Each entry is scored on a 4-point scale, with higher scores indicating higher adherence to functional exercise. The scale has good reliability and validity and can fully reflect the compliance of functional exercise in stroke patients. The total score of the scale is the sum of the scores of each item. The Cronbach’s alpha of the scale in this study was 0.942.

#### 2.2.4. Tampa scale of kinesiophobia

Tampa scale of kinesiophobia (TSK-11), designed by Woby et al. (2005) in the UK, mainly detects kinesiophobia in patients with chronic pain. In the Chinese version developed by Cai et al. (2019). The Chinese version included three dimensions (activity cognition, activity behavior, and activity attitude) and 11 items. The Likert 5-point scale was used (not at all = 1 to strongly agree = 4). The total scores ranged from 11 to 44. At the same time, Cronbach’s alpha of TSK-11 for the present study was 0.963.

#### 2.2.5. Motivation in stroke patients for rehabilitation scale

The scale was developed by Japanese experts Yoshida et al. (2022), in its original version in English, and was mainly used to measure how stroke patients were motivated to recover in the rehabilitation ward. Tan et al. (2023) translated this scale into Chinese and validated the reliability of the Chinese version of MORE. The Cronbach’s alpha coefficient of the Chinese version of the MORE scale was 0.983. The scale was one-dimensional, with 17 items. The scale was scored on a 7-point Likert scale.

### 2.3. Data collection

This study was conducted by means of questionnaire distribution. The consent of the relevant hospital officials was obtained before the survey, and the questionnaire was distributed by the researcher himself. Before the questionnaires were distributed, the purpose, significance, and precautions of this study were explained to the study participants, and the anonymity of the questionnaires was ensured. A total of 356 questionnaires were distributed in this study and 350 valid questionnaires were recovered, with a valid recovery rate of 98.31%.

### 2.4. Statistical analysis

This study used SPSS 26.0 software for data analysis. Descriptive statistics were used to understand the current status of perceived social support, exercise adherence, kinesiophobia, and motivation for rehabilitation in elderly stroke patients. ANOVA analysis or t-test was used to further explore the influential factors affecting motivation for stroke rehabilitation. The results of the correlation analysis were completed and displayed by R (V4.0.2). Pearson correlation analysis was performed using the R (V4.0.2) corrplot package for PSSS, EAQ, TSK, and MORE indicators, which were significantly correlated at *p* < 0.05 when *R* > 0.5 or *R* < −0.5. The demographic variables that were statistically significant in the ANOVA analysis, PSSS, EAQ, and TSK were included in the linear regression analysis to derive the factors influencing motivation for rehabilitation in stroke patients. *p* < 0.05 means the difference is statistically significant.

### 2.5. Ethics approval and consent to participate

Each patient willingly enrolled, gave informed consent to the study, and signed an informed consent form, and their anonymity was preserved. The Declaration of Helsinki was adhered to during this study. The study was approved by the Ethics Committee of Jinzhou Medical University (NO. JZMULL2022008).

## 3. Results

### 3.1. General demographic data and ANOVA or *t*-test analysis of rehabilitation motivation in older adult patients with stroke

The 350 participants with stroke included 178 male participants (50.9%) and 172 female participants (49.1%). 21.4% of patients were 60–69 years old; 35.1% of patients were 70–79 years old; 43.4% were aged ≥80 years. Overall characteristics can be found in Table 1.

**Table 1:** General demographic information and ANOVA or *t*-test analysis of motivation for rehabilitation (*n* = 350).

Characteristics	*n*	%	MORE( $\bar{x}$ ±s)	t/*F*	*p*
Sex					
Male	178	50.9	65.86 ± 24.75	0.441	0.660
Female	172	49.1	64.65 ± 26.76		
Age (years)					
60–69	75	21.4	67.43 ± 27.38	0.910	0.691
70–79	123	35.1	64.81 ± 26.19		
≥80	152	43.4	64.56 ± 24.60		
Education					
Junior	73	20.9	59.08 ± 24.70	1.263	0.084
Middle	139	39.7	65.30 ± 27.64		
High school	80	22.9	70.78 ± 23.72		
College and above	58	16.6	65.36 ± 23.68		
Time of stroke					
<6 months	102	29.1	68.85 ± 24.54	1.598	0.003
6 ~ 12 months	151	43.1	65.76 ± 25.86		
≥1 years	97	27.7	60.71 ± 26.32		
Lesion location					
Left	109	31.1	62.48 ± 27.21	1.468	0.012
Right	125	35.7	73.38 ± 24.89		
Both	116	33.1	59.12 ± 22.99		
Type of stroke					
Hemorrhage	191	54.6	63.86 ± 25.54	−1.120	0.264
Ischemic	159	45.4	66.95 ± 25.93		
Medical insurance					
Yes	284	81.1	66.32 ± 26.21	1.604	0.110
No	66	18.9	60.70 ± 23.16		

### 3.2. Score of PSSS, EAQ, TSK-11, and MORE of the stroke patients

The mean (SD) scores for PSSS were (49.66 ± 18.25), the mean (SD) scores for EEAQ were (37.02 ± 9.30), the mean (SD) scores for TSK-11 were (29.54 ± 9.33), and the mean (SD) scores for MORE were (65.26 ± 25.73). The score of other dimensions among stroke patients was shown in Table 2.

**Table 2:** Scores on the PSSS, EAQ, TSK-11, and MORE (*n* = 350， $\bar{x}$ *±s*).

Scale	Projects	Items	Score	Entry parity score
PSSS	Total score	12	49.66 ± 18.25	4.14 ± 1.52
	Family support	4	16.59 ± 6.18	4.15 ± 1.52
	Friend support	4	16.64 ± 6.22	4.16 ± 1.55
	Other support	4	16.41 ± 6.13	4.10 ± 1.53
EAQ	Total score	14	37.02 ± 9.30	2.64 ± 0.66
	Physical exercise adherence	8	21.27 ± 5.54	2.66 ± 0.69
	Exercise monitoring adherence	3	7.99 ± 2.17	2.66 ± 0.72
	Active advice seeking adherence	3	7.77 ± 2.20	2.59 ± 0.73
TSK-11	Total score	11	29.54 ± 9.33	2.69 ± 0.85
	Activity behavior	3	15.69 ± 4.03	2.71 ± 0.92
	Activity awareness	6	16.11 ± 5.12	2.68 ± 0.85
	Activity attitude	2	5.31 ± 1.81	2.66 ± 0.91
MORE	Total score	17	65.26 ± 25.73	3.84 ± 1.51

PSSS, Multidimensional Scale of Perceived Social Support; EAQ, Questionnaire of Exercise Adherence; TSK-11, Tampa scale of Kinesiophobia; MORE, Motivation in Stroke Patients for Rehabilitation scale.

### 3.3. The correlation between perceived social support, exercise adherence, kinesiophobia, and motivation for rehabilitation in older adult patients with stroke.

**Figure 1:**
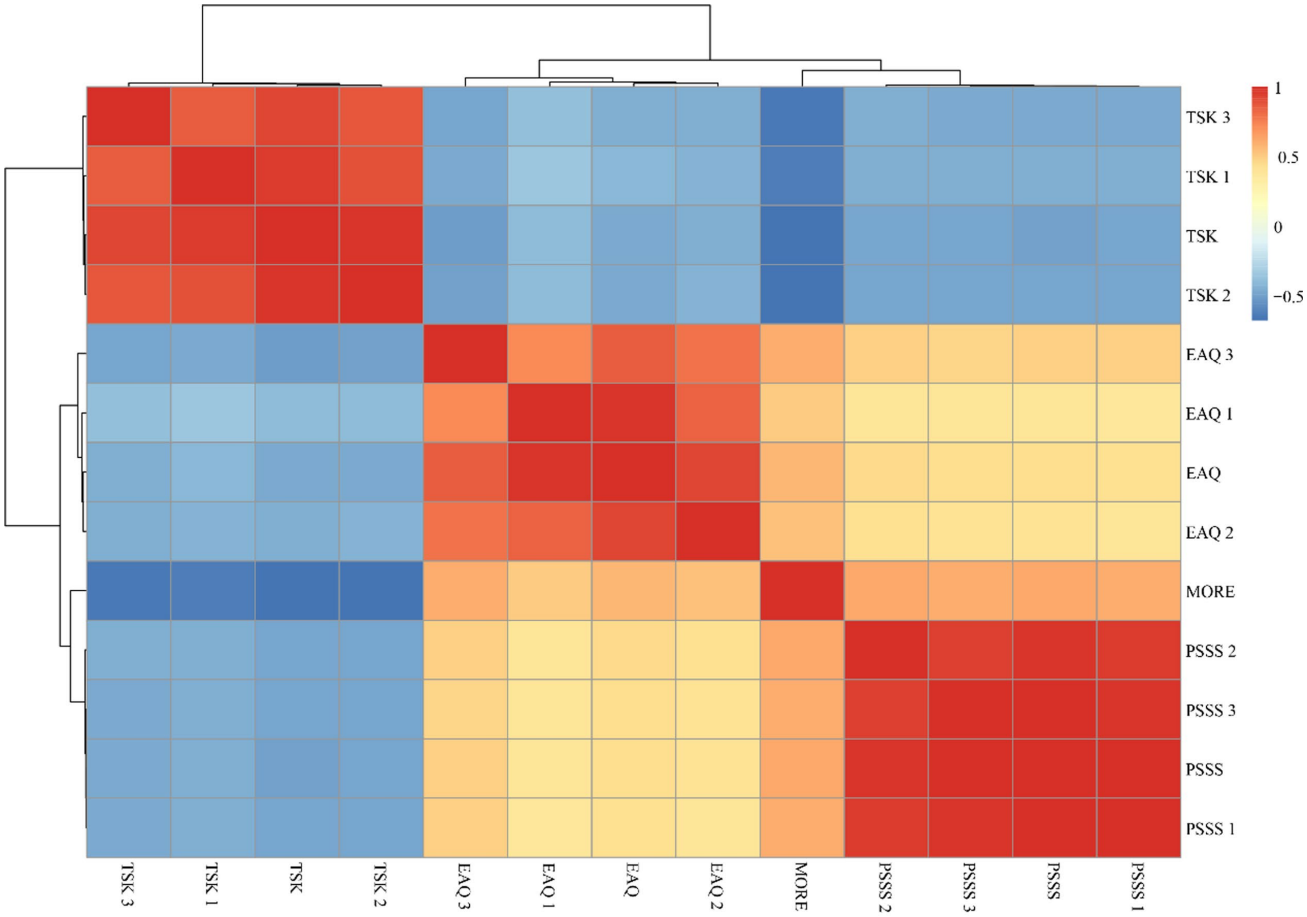
Correlation analysis of perceived social support, exercise adherence, kinesiophobia, and motivation for rehabilitation in older adult patients with stroke. PSSS: perceived social support total score; PSSS 1: family support; PSSS 2: friend support; PSSS 3: other support; EAQ: exercise adherence total score; EAQ 1: physical exercise adherence; EAQ 2: exercise monitoring adherence; EAQ 3: active advice seeking adherence; TSK: kinesiophobia total score; TSK 1: activity behavior; TSK 2: activity cognitive; TSK 3: activity attitude; MORE: total stroke rehabilitation motivation score.

Apprehending social support, exercise adherence, and motivation to recover were positively correlated (*r* = 0.619, *p* < 0.01; *r* = 0.569, *p* < 0.01), and kinesiophobia was negatively correlated with motivation to recover (*r* = −0.677, *p* < 0.01) (Figure 1).

### 3.4. Effect of demographic statistical data on MORE

Patients’ motivation to recover was used as the dependent variable, and the statistically significant variables in the ANOVA analysis (time to stroke and location of the lesion) were used as the first level of independent variables, and PSSS, EAQ, and TSK were used as the second level of independent variables, and stratified regression analysis was selected. See Table 3 for the assignment method of independent variables The results showed that PSSS, EEAQ, and TSK had significant effects on motivation to recover, explaining 60.7% of the total variance in motivation to recover. (Table 4).

**Table 3:** Assignment of independent variables.

Independent variable	Assignment method
Time of stroke	<6 months = 1，6 ~ 12 months = 2, ≥1 years = 3
Lesion location	Left side = 1，Right side = 2, Bilateral = 3
PSSS	Original value
EAQ	Original value
TSK-11	Original value

PSSS, Multidimensional Scale of Perceived Social Support; EAQ, Questionnaire of Exercise Adherence; TSK-11, Tampa scale of Kinesiophobia.

**Table 4:** Results of linear regression analysis of factors associated with MORE in older adult patients with stroke.

Number of layers	Factors	Regression coefficient	Standard error	Standardized regression coefficient	*t*	*P*	*R* ^2^	Adjusted *R*^2^	*F*	*p*
The first layer	(constant)	64.43	3.234	–	19.921	0.000	0.072	0.062	6.725	0.000
	Time of stroke	−7.104	3.544	−0.124	−2.005	0.046				
	Lesion location	11.273	3.302	0.210	3.414	0.001				
The second layer	(constant)	52.90	7.082	–	7.469	0.000	0.615	0.607	78.091	0.000
	PSSS	0.419	0.057	0.287	7.333	0.000				
	EAQ	0.648	0.110	0.233	5.866	0.000				
	TSK-11	−1.121	0.113	−0.407	−9.909	0.000				

PSSS, Multidimensional Scale of Perceived Social Support; EAQ, Questionnaire of Exercise Adherence; TSK-11, Tampa scale of Kinesiophobia; MORE, Motivation in Stroke Patients for Rehabilitation scale.

## 4. Discussion

The current study used a cross-sectional survey focusing on the degree of motivation for rehabilitation and the influencing factors affecting patients’ motivation for rehabilitation among current Chinese stroke patients. The results of the study showed that among 350 stroke patients, the rehabilitation motivation score was (65.26 ± 25.73), which was at an intermediate level. One of the reasons for this may be the decline in physical adaptability of older adults as they age, with declining immunity, declining physiological function, and organ decline. The second may be that the patients’ somatic body is damaged by the disease, and factors such as reduced somatic mobility and unstable gait make the patients’ own sense of belief in rehabilitation lower and their motivation for rehabilitation low.

Therefore, during the rehabilitation of older adults with stroke, the rehabilitation work is difficult and intensive, the overall rehabilitation needs of older adults with stroke are high, and active psychological guidance and rehabilitation guidance should be provided to patients during medical care (Kobylańska et al., 2018; Janssen et al., 2020). Therefore, it is necessary to analyze the motivation and influencing factors of rehabilitation in older adults with stroke and use them as an important basis for guiding and helping patients to recover.

The stratified regression results showed that when PSSS, EAQ, and TSK were placed in the second stratum, the adjusted R^2^ increased from 6.2 to 60.7% and the degree of explanation of the regression equation for stroke rehabilitation motivation increased by 54.5%. This suggests that the stronger the level of motivation for rehabilitation in elderly stroke patients, the higher the social support received by individuals, the better their own exercise compliance, and the lesser the level of agoraphobia, which is more conducive to later rehabilitation. In the above study, the time of stroke, location of the lesion, perceived social support, exercise adherence, and kinesiophobia influenced the patients’ motivation to recover.

The duration of stroke is an influential factor affecting the motivation for rehabilitation in elderly stroke patients, which is consistent with the findings of Kobylańska et al. (2018). As the duration of illness lengthens, patients perceive previous rehabilitation exercises as ineffective and with no significant improvement in functional impairment, leading to a gradual decrease in their sense of belief in rehabilitation and a weakening of motivation to rehabilitate (Ytterberg et al., 2020). Medical and nursing staff should focus on elderly stroke patients with longer disease duration, give positive psychological hints for rehabilitation, and encourage patients to adhere to exercise and improve their quality of life.

Studies have shown that the location of the stroke lesion affects the level of motivation to recover. Patients whose lesions were bilateral were more motivated to recover, which is consistent with the findings of Petruseviciene and Krisciūnas (2008). When the patient’s condition is involved bilaterally, the patient has an active and urgent need for rehabilitation, is able to listen carefully to the rehabilitation advice given by the physician, and adheres to effective exercise activities, thus demonstrating a high level of motivation for rehabilitation. Therefore, healthcare professionals should observe patients’ negative attitudes toward rehabilitation early in their work, make them aware of the importance of rehabilitation exercises for their future lives, and guide them to positive rehabilitation intentions.

Studies have shown that perceived social support is positively correlated with motivation to recover, and the higher the perceived social support of stroke patients, the stronger their motivation to recover and the more likely they are to contribute to a lower disability rate, which is consistent with the findings of Lee et al. (2021) and Darsin Singh et al. (2022). The reason for this is that older patients receive adequate medical coverage through family, friends, health care providers, and society to build confidence in overcoming the disease (Lobo et al., 2021) and are able to follow the rehabilitation advice given by health care providers and follow the plan, which in turn increases the patients’ own motivation to rehabilitate. A low level of motivation to recover delays the recovery of all functions and increases the burden on society and the family (Lin et al., 2019).

Studies have shown that exercise adherence is positively correlated with motivation for rehabilitation in stroke patients, and the higher the patients’ own exercise compliance, the higher their degree of motivation for rehabilitation, which is consistent with the findings of Das et al. (2021) and Petersen et al. (2020). This may be due to the fact that older people have a high level of trust in health care professionals, are able to follow the advice given by professionals, follow the prescribed program, and have a high level of exercise compliance, which in turn leads to an increase in their motivation for rehabilitation. As stroke causes neurological impairment, the more severe the impairment, then the worse the patient’s ability to move autonomously (Li et al., 2022). Therefore, healthcare professionals should guide patients in rehabilitation training to help elderly stroke patients to better recover their body functions, improve their quality of life level, return to their families, and reintegrate into society.

The degree of kinesiophobia in stroke patients was negatively correlated with motivation for rehabilitation, i.e., the higher the patient’s kinesiophobia, the lower the degree of motivation for rehabilitation, which is consistent with the findings of Naseri et al. (2020) and Sever et al. (2022). Analyzing the reasons for this, it is possible that the patients’ decreased physical function and limited social activities increase their fear of exercise, which in turn leads to a decrease in their beliefs about rehabilitation and a decrease in motivation. Kinesiophobia is a risk factor for motor rehabilitation, which will cause patients to experience adverse emotions and respond negatively to the treatment and rehabilitation of the disease (Baykal Şahin et al., 2021). Low levels of kinesiophobia are more inclined to opt for positive concepts that will make patients interested in exercise and able to adhere to it according to their personal abilities and personal rehabilitation plans (Conway et al., 2022).

## 5. Limitations

However, there are some limitations to this study. First, this study was conducted only in the northern region of China, which may be somewhat regional in nature. Second, the sample size in the study was limited and not representative of the overall level, and future research could be conducted on a large scale. Third, only cross-sectional surveys were used in this study, and no intervention studies have been conducted.

## 6. Conclusion

In this study, we found that Chinese stroke patients currently have an intermediate level of motivation for rehabilitation, but they also have slightly higher levels of kinesiophobia. Our study proposes to improve stroke patients’ motivation for rehabilitation while also focusing on their perceived social support, exercise adherence, and kinesiophobia levels. To establish a sound social and medical service system and focus on the elderly group to improve the quality of life of stroke patients.

## Data availability statement

The raw data supporting the conclusions of this article will be made available by the authors, without undue reservation.

## Ethics statement

The studies involving human participants were reviewed and approved by Ethics Committee of Jinzhou Medical University (No. JZMULL2022008). The patients/participants provided their written informed consent to participate in this study. Written informed consent was obtained from the individual(s) for the publication of any potentially identifiable images or data included in this article.

## Author contributions

MT and HL study concept and design. MT implementation. MT and XW statistical analysis and writing the first draft. All authors contributed to the article and approved the submitted version.

## Funding

This study was supported by the China Association of Gerontology and Geriatrics, No. 2021-04-01.

## Conflict of Interest

The authors declare that the research was conducted in the absence of any commercial or financial relationships that could be construed as a potential conflict of interest.

## Publisher’s note

All claims expressed in this article are solely those of the authors and do not necessarily represent those of their affiliated organizations, or those of the publisher, the editors and the reviewers. Any product that may be evaluated in this article, or claim that may be made by its manufacturer, is not guaranteed or endorsed by the publisher.
